# The Minds of Nations

**Published:** 1894-02-24

**Authors:** 


### THE STUDY OF MAN.

### V.—THE MINDS OF NATIONS.

It has been frequently observed, and is the principal difficulty which besets the historian and critic of the study of man, that the subject of his investigation is not, as in other cases, one set of causes to be traced through their varying phenomena, but rather one species, the various characteristics and behaviour of which form part of the subject matter of very different sciences, and must in each aspect be examined by an appropriate method, without immediate reference to the other qualities with which it is associated in the nature of things.

On its anthropological side, for example, the study of man is a special branch of zoology; but in its ethnological bearings it comes closely into connection with mental, moral, and in the largest sense with historical and political science. There is a sense, indeed, in which all these are but special and highly elaborate departments of it; but it will be convenient for our present purpose to consider them only in so far as they throw direct light upon the central problems of the origin, the essence, and the development of humanity.

With psychology in particular we have here very little to do, though there are certainly points of general anthropology on which the mental development and habits of the individual man offer valuable evidence. But it was not till a wider view came to be held of the scope of mental science that the ethnographical data which had long been accumulating could be interpreted by its methods and principles. It is true that very early expressions can be found of the conviction that man is essentially a social being, and that his distinctive character cannot be fully or truly appreciated unless he is regarded as a member in a community of his like. Herder in particular in the last century protests very strongly, in his "Ideen zur Gesehichte der Menschbeit," against the tradition of an actually unsocial state of man, and insists on the study of the general characteristics of the subdivisions of the species as an aid to the comprehension of the nature of mankind as a whole. And the poet Schiller goes further, and suggests that the so-called lower races stand in relation to the higher, as children to adults; being, in fact, one of the first to formulate the principle of the interpretation of phylogeny through ontogeny; of the secular modification of the species, through its recapitulation in the life history of the individual, which is of so vital importance in recent biological work.

Curiously enough, the conception of what has come to be called the psychology of national life, the least exact and most widely speculative branch of the study of mind, seems to be due to the founder of a psychological system which is formulated with almost mathematical precision. For it is in Herbart's "Lehrbuch," published in 1815, that the suggestion of a Völker psychologie first definitely occurs, and the earliest and most consciously systematic exponents of it are all closely associated with the Herbartian school.

Theodor Waitz, the first and most important of them, was born in 1821, and after study under Drobisch, at Leipzig, and preliminary excursions into Aristotelian philosophy and scholarship, published in 1849 his "Lehrbuch der Psychologie als Naturwissenschaft," which makes a decided advance in psychological method, and indicates already the principles which underlie his subsequent work. Of his great book, "Die Anthropologie der Naturvölker," four volumes appeared between 1859 and his death in 1864; and two more were published by his friend Gerland in 1865 and 1872. They contain accurate and detailed accounts of the physical and intellectual character, of the mode of life, the manners, usages, organisations, traditions, and beliefs of nearly every uncivilised or partly civilised people, verified and attested with references which prove the vast erudition and unwearied industry of the author. This great accumulation of classified evidence, not unrelieved by cautious and acute interpretations, was conceived as the basis for a philosophy of religion which was never realised. The first volume, which is largely occupied with discussions of the unity of mankind, and the variability of races, had the misfortune to be superseded almost at once by the "Origin of Species"; but the facts contained in this, and still more in succeeding volumes, are a collection of the greatest value, and sufficiently illustrate the principles of the writer, and the possibility of establishing general psychological laws, to explain the habits and observances of human societies.

In the same year, 1859, appeared also the first volume of the "Zeitschrift fur Volkerpsychologie und Sprachwissenschaft," edited by Lazarus and Stendhall, and supported by such various authorities as Delbrück, Cohen, and Lange, the author of the "History of Materialism." Its programme is very clearly set forth in the introduction to the first number, and in the leading essays in the succeeding volumes.

Take a single tree, or a single cell of the yeast plant. It is simply a subject of classificatory, or morphological, or physiological botany. But plant ten thousand trees, or throw a handful of yeast into a vat; they have not ceased to be botanical specimens, but in the aggregate they are the subject matter of new sciences—and arts besides—of forestry, or brewery, as the case may be. And so with human aggregates. We cannot confine ourselves to the study of the individual; but find a new and larger life in the "social organism," and new laws which express its relations to the collective environment. It is the old analogy between the physical body and the "body politic," carried out in this instance with much spirit and great wealth of every kind of material, as a glance at the indices of the "Zeitschrift" will show, and, it must be confessed, with very adequate provision as a rule against the fallacies which have always beset the theory; but, at the same time, without the systematic fruit as yet which at first sight the method seemed to promise. A particularly notable feature is the inclusion of the study of language in the scope of the psychology of nations, a source of evidence which we shall have to consider later on by itself.

Even more thoroughgoing are the rather earlier contributions of Comte, Quetelet, and Klemm. Klemm even set up in opposition to the physical anthropologists, a classification of mankind into an active, progressive, and spiritual, and a passive conservative and formalist variety, and tried to bring the principal physical characteristics—cephalic and orbital indices, complexion, hair, and features—into harmony with this distinction. Quetelet combined an empirical with a mathematical point of view, and was particularly successful in the application of his theory of averages to the phenomena of social and economic life. His treatise of 1848, "*Du Systeme Sociale et des Lois qui le régissent,*" has found numerous amplifications; in particular Schäffle's "*Ban und liben des sozialen Körper's*" (1875), which takes up the position that "the spiritual life of the social body is 'a higher power of the spiritual life of the individual'" (I. S.); and the "*Gedanhen über die sozial wissenschaft der Zerhunft*" of Paul von Lilienfeld (1873).

Comte's work is best paralleled in England by the "Sociology" of Herbert Spencer, and by the invaluable work in the wide field of religion and general culture, of Tyler, Bastian, and Ratzel, and of Taine and Post in the special applications of the comparative ethnological method to the philosophy of literature and of law. J. L. M.

